# Investigation of the Effect of Temperature on the Structure of SARS-CoV-2 Spike Protein by Molecular Dynamics Simulations

**DOI:** 10.3389/fmolb.2020.583523

**Published:** 2020-10-16

**Authors:** Soumya Lipsa Rath, Kishant Kumar

**Affiliations:** ^1^Department of Biotechnology, National Institute of Technology Warangal, Warangal, India; ^2^Department of Chemical Engineering, National Institute of Technology Warangal, Warangal, India

**Keywords:** structural protein, receptor binding motif, N-terminal domain, closed conformation, temperature-sensitive

## Abstract

Statistical and epidemiological data imply temperature sensitivity of the SARS-CoV-2 coronavirus. However, the molecular level understanding of the virus structure at different temperature is still not clear. Spike protein is the outermost structural protein of the SARS-CoV-2 virus which interacts with the Angiotensin Converting Enzyme 2 (ACE2), a human receptor, and enters the respiratory system. In this study, we performed an all atom molecular dynamics simulation to study the effect of temperature on the structure of the Spike protein. After 200 ns of simulation at different temperatures, we came across some interesting phenomena exhibited by the protein. We found that the solvent exposed domain of Spike protein, namely S1, is more mobile than the transmembrane domain, S2. Structural studies implied the presence of several charged residues on the surface of N-terminal Domain of S1 which are optimally oriented at 10–30°C. Bioinformatics analyses indicated that it is capable of binding to other human receptors and should not be disregarded. Additionally, we found that receptor binding motif (RBM), present on the receptor binding domain (RBD) of S1, begins to close around temperature of 40°C and attains a completely closed conformation at 50°C. We also found that the presence of glycan moieties did not influence the observed protein dynamics. Nevertheless, the closed conformation disables its ability to bind to ACE2, due to the burying of its receptor binding residues. Our results clearly show that there are active and inactive states of the protein at different temperatures. This would not only prove beneficial for understanding the fundamental nature of the virus, but would be also useful in the development of vaccines and therapeutics.

## Graphical Abstract

Closure of the receptor binding motif of SARS-COV-2 Spike protein at high temperatures.

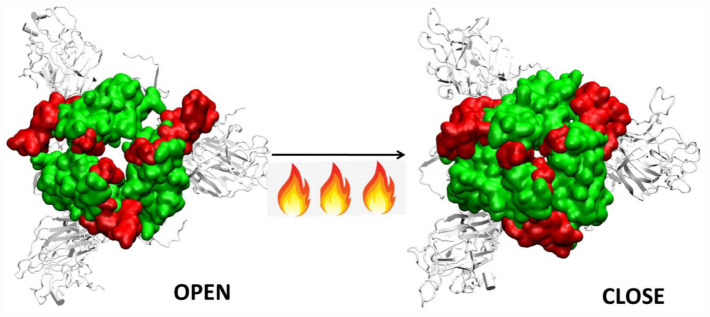

## Introduction

Severe Acute Respiratory Syndrome Coronavirus 2 or SARS-COV-2, attacks the cells of the human respiratory system. Recent studies have found that the virus also interacts with the cells of the digestive system, renal system, liver, pancreas, eyes and brain (Gordon et al., 2020). It is known to cause severe sickness and is fatal in many cases (World Health Organization [WHO], 2020). It is believed that the virus originated in bats, which act as the natural reservoir; subsequently it got transmitted to human. It then gradually spread across almost all the nations through aerial transmission resulting in one of the worst known global pandemic of this century (MacKenzie and Smith, 2020).

SARS-COV-2 is one of the seven forms of coronaviruses that affect the human population. The other known coronaviruses include HCoV-229E, HCoV-OC43, SARS-CoV, HCoV-NL63, HCoV-HKU1, and MERS-CoV (Gao et al., 2016; Su et al., 2016). Their infection varies from common cold to SARS, MERS or Covid19 (Su et al., 2016). These viruses have been observed to affect the human population predominantly during a particular season. For instance, the 2002 SARS infections began during the cold winters of November and after 8 months, the number of reported cases became almost negligible (Su et al., 2016). Statistics show that countries with hot and humid weather conditions had lesser number of infectious cases of SARS (Chan et al., 2011). However, MERS-COV, which was identified in Middle East regions, affected individuals during the summer (Su et al., 2016). Thus, the disease epidemiology suggests that the virus is found to be prominent in certain climatic conditions only.

The viability of SARS-COV-2 was measured on different surfaces by Chin et al. (2020), who found that the virus droplets survived at 4°C but quickly deactivated at elevated temperatures of 50°C. Smooth surfaces, plastics and iron show greater viability of the virus compared to that of paper, tissue, wood or cloth. Surgical masks had detectable viruses even on 7th day (Casanova et al., 2010; Van Doremalen et al., 2020). Soaps and disinfectants which disintegrate the virus membrane and structural proteins are a potent example of how the modulation of atmospheric conditions can affect the virus viability. Statistical reports by Cai et al. (2007), and several others had shown that tropical countries like Malaysia, Indonesia or Thailand with high temperature and high relative humidity did not have major community outbreaks of SARS (Tan et al., 2005; Chan et al., 2011). Although viruses cannot be killed like bacteria by autoclaving, temperature sensitivity of virus have been reported several times in the past. Seasonal Rhinoviruses could not replicate at 37°C, whereas 33–35°C is ideal for their survival in nasal cavity (Foxman et al., 2015). Influenza was found to be effective at a temperature around 37°C, whereas higher temperatures of 41°C resulted in clumping of viruses on cell surfaces (Ishida et al., 2002; Pelletier et al., 2011; Lowen and Steel, 2014). Similarly, the viability of SARS virus that persisted for 5 days at temperatures ranging between 22–25°C and 40–50% humidity, was lost when the temperature was raised to 38°C and 95% humidity (Chan et al., 2011).

**FIGURE 1:**
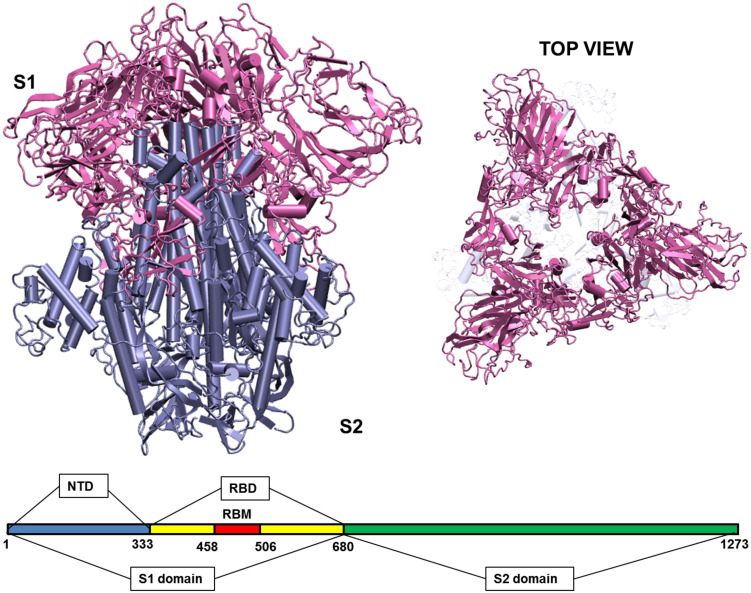
Structure of the Spike glycoprotein. The Spike glycoprotein, S1 and S2 domains, in the absence of glycan residues and lipid bilayer. The S1 domain is shown in pink and S2 in iceblue color. The top view of the protein shows a triangular arrangement of the S1 domain. Below the structure is a schematic showing the location of important regions on the S1 and S2 domains of the protein. The abbreviations in the S1 and S2 domains are: – NTD, N-terminal domain; RBD, receptor binding domain; RBM, receptor binding motif. The starting and ending residues are numbered.

When the virus is exposed to different temperature conditions, the initial interactions of the atmosphere occur with the structural proteins. There are four major structural proteins present on the virus, the Spike glycoprotein, the Envelope protein, the Membrane protein and the Nucleocapsid. Each of the proteins performs specific functions in receptor binding, viral assembly and genome release (Astuti and Ysrafil, 2020). One of the first and largest structural proteins of the Coronavirus is the Spike glycoprotein (Li, 2016). The protein exists as a homotrimer where each monomer consists of 1,273 amino acid residues (Figure 1) and is intertwined with each other. Each monomer has two domains, namely S1 and S2 (Walls et al., 2020). The S1 and S2 domains are cleaved at a furin site by a host cell protease (Belouzard et al., 2009; Walls et al., 2020). The S1 domain lies predominantly above the lipid bilayer. The S2 domain, which is a class I transmembrane domain, travels across the bilayer and ends toward the inner side of the lipid membrane (Walls et al., 2020). Figure 1 shows the two domains of the Spike glycoprotein.

The S1 domain comprises of mostly beta pleated sheets. It can be further classified into Receptor Binding domain (RBD) and N-terminal Domain (NTD). The RBD binds to Angiotensin Converting Enzyme 2 (ACE 2) on the host cells (Wang et al., 2020). It lies on the top of the complex, where around 14 residues from the RBD domain bind to the ACE2 receptor on the host protein (Yuan et al., 2017; Lan et al., 2020). The NTD is the outermost domain that is relatively more exposed and lies on the three sides giving a triangular shape to the protein when viewed from top (Figure 1). The NTD has a galectin fold and is known to bind to the sugar moieties (Yuan et al., 2017). The S2 domain on the other hand is a transmembrane region with strong interchain bonding between the residues. It is mostly α-helical and forms a triangle when viewed from bottom, though there is no overlapping of the top and bottom triangles.

Temperature is a very significant variable parameter for proteins because proteins respond differently in high and low temperature conditions. Many proteins have high thermal stability while others can unfold or even denature at high temperatures (Dong et al., 2018; Julió Plana et al., 2019). Experimental studies by Xiong et al. (2020), where they generated a mutated Spike protein that exhibited high level of thermal stability. Further studies had shown that the activity of the SARS-CoV-2 reduced significantly at high temperatures, when compared to that of SARS-CoV. It was concluded that the comparatively lower energy barrier of SARS-CoV-2 would result in higher transmission rate of the virus (Ou et al., 2020). MD studies also spoke about the temperature sensitivity of RBD of the SARS-CoV-2 compared to its predecessor SARS-CoV (He et al., 2020). During November, 2019, when the first outbreak of Covid19 was reported, the temperature in Wuhan, China was around 17°C in the morning and 8°C at night. Tropical countries such as India, where a large number of cases still persist, had over 40°C of temperature (Bukhari and Jameel, 2020; Wu et al., 2020). Although statistical and experimental evidence show that temperature influences the activity and virulence of the virus, we still lack the understanding of the molecular level changes that are taking place in the virus due to the different weather conditions. Till date, there is no concrete evidence on whether atmospheric conditions actually influence the structure of the virus.

Here, by using all atom molecular dynamics (MD) simulations we explore the dynamics of the Spike glycoprotein of SARS-COV-2 at different temperatures. This is the first molecular study on the environmental influence on the protein structure. Results suggest that S1 domain is more flexible than S2. In the S1 domain, we observed the sensitivity of the receptor binding motif to different temperatures. We also found that the N-terminal domain of the protein has the potential of binding to different human receptors. The study will not only help us in understanding the nature of the virus but is also useful to design effective therapeutic strategies.

## Materials and Methods

The complete model of the Spike glycoprotein of SARS-COV-2 was obtained from Zhang lab (GenBank: QHD43416.1). This model was considered because it had modeled the missing 871 residues that were absent in the crystal structure (PDB ID 6VXX). It had a Template Modeling (TM) score of 0.6 (Xu and Zhang, 2010). The initial Root Mean Square Deviation (RMSD) between the model and the closed crystal structure of Spike glycoprotein (PDB ID 6VXX) was found to be 1.54 Å. The model was devoid of N-acetyl glycosamine (NAG) glycan residues and consisted of the glycoprotein trimer where each monomer had amino acids ranging from 1 to 1,273. The structure was initially solvated with a TIP3P water box having a cubic box of size 17.9 × 17.9 × 17.9 nm and 569,293 atoms with water and ions (Jorgensen et al., 1983). The minimum distance between the protein and the edge of the water box was fixed at 13 Å. Particle-Mesh Ewald (PME) method was used for electrostatic interactions using a grids pacing of 0.16 nm and a 1.0 nm cutoff. After energy minimization and equilibration, by maintaining harmonic restraints on the protein heavy atoms, the system was heated to 300 K in a canonical ensemble. The harmonic restraints were gradually reduced to zero and solvent density was adjusted under isobaric and isothermal conditions at 1 atm and 300 K. This was followed by 500 ps NVT and 500 ps NPT equilibration with harmonic restraints of 1,000 kJ mol^–1^ nm^–2^ on the heavy atoms. Production run for all the systems was carried out for 200 ns till it reached a stable RMSD. All simulations were carried out in GROMACS (2020) with AMBERff99SB-ILDN force field for proteins (Lindorff-Larsen et al., 2010; GROMACS, 2020). The long-range electrostatic interactions were treated by using Particle-Mesh Ewald sum and SHAKE was used to constrain all bonds involving hydrogen atoms. After equilibration, systems were heated or cooled at different temperatures (Supplementary Table S1) and simulated for 200ns. All analyses were carried out using Gromacs analysis tools (Lindorff-Larsen et al., 2010). Protein Blast was used to search similar sequences in the human proteome. The Blast Tree View widget helped us generate the phylogenetic tree which is a simple distance based clustering of the sequences based on pairwise alignment results of Blast relative to the query sequence (Sayers et al., 2009). VMD was used for visualization of results and generation of figures (Humphrey et al., 1996). Principal component analysis was carried out in Gromacs. Pymol was used for generation of porcupine plots (The PyMOL Molecular Graphics System, 2019). We used the glycan bound Spike protein deposited by Woo et al. (2020) in CHARMM GUI as starting structures, for understanding the difference in dynamics of Spike protein in the presence of carbohydrates (Jo et al., 2008).

## Results and Discussion

The crystal structure of the Spike glycoprotein (PDB: 6VXX) was found to have 871 missing residues. Thus, for our study we considered the complete model of the trimeric Spike protein generated by Xu and Zhang (2010) and had a Template modeling score of 0.6. The model was devoid of N-acetyl glucosamine (NAG) sugar moieties which are known to bind and stabilize the protein. The envelope lipid bilayer was not considered in the work to avoid large system size in atomistic simulations. After initial minimization and equilibration, we generated five different systems having temperatures ranging from 10 to 50°C at an interval of 10 degrees. This was done to maintain the uniformity of the simulations, where temperature was the only variable that was different. It should be noted that when a temperature is raised, the electronic distribution of the atoms undergo change. However, classical force fields like AMBER-ff99SB-ILDN are based on certain approximations and the changes in temperature in a particular force field do not impact the results very significantly (Lindorff-Larsen et al., 2013). In addition, a temperature of 70°C was also imposed on the system to observe any possible deformation in the structure of spike protein, although this high temperature is not realistic to imitate the environmental condition (Supplementary Table S1). Production run for 200 ns was carried out in isothermal isobaric (NPT) ensemble. To understand the impact of glycans on the dynamics, subsequently three additional simulations were run at 10, 30, and 50°C.

### Spike Glycoproteins Are Sensitive to Temperature

After performing 200 ns of classical Molecular dynamics simulations, the root mean square deviation (RMSD) of the trajectory, with respect to the starting structure, was calculated to check if the systems have attained stability. Supplementary Figure S1 shows the complete RMSD of all the systems at different temperatures. It can be seen that the stability was attained within the first 50 ns of the simulation time, thus, indicating that the systems are well equilibrated. The RMS values lie between 0.6 and 0.7 nm for all the systems with an exception at 40°C where a marginally higher RMSD was seen after 100 ns of simulation time. At temperatures 20 and 30°C, a small rise in RMSD curves after 100 ns of simulation time was observed. This implies that the Spike protein was more stable at temperatures 10 and 50°C. The overall energy of the systems were monitored and it was also found to have attained stability (data not shown).

**FIGURE 2:**
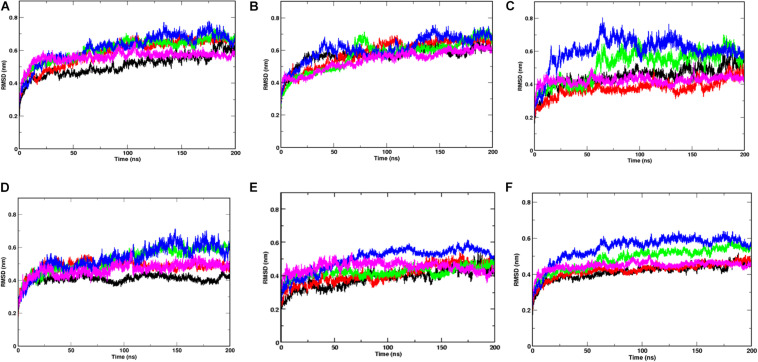
Temperature sensitivity of the S1 domain of Spike protein. RMSDs of Spike glycoprotein **(A)** S1 and **(B)** S2 domain showing stability of the S2 chains. The differential fluctuations of chains **(C)** A, **(D)** B, **(E)** C, and the average RMSD of the three chains **(F)** of S1 domain at 10°C (black), 20°C (red), 30°C (green), 40°C (blue), and 50°C (magenta) implying effect of temperature of the chain stability.

Since, the protein comprises of two distinct domains S1 and S2, we checked the RMSD of S1 and S2 domains individually, with respect to the starting structure, to understand the cause for higher RMSD values observed at 20, 30, and 40°C (Figure 2). The RMS values of S1 domain at 20, 30, and 40°C were found to be around 0.7 nm, nearly 0.5 nm more than simulations at 10 and 50°C, respectively. A similar trend was observed in the RMSD of S2 domain, but, the difference in values was only 0.15 nm. Although, in this study, we haven’t considered the bilayer lipid membrane of the SARS-COV-2 envelope inside which the Spike glycoprotein resides, the S2 domain shows remarkable stability in its RMSD values (Figure 2). The stability of the S2 domain can be conferred to the strong interchain interactions among the highly α-helical S2 domain.

Since the Spike protein is a homotrimer, the S1 of individual domains was checked to account for the difference in fluctuations. Figures 2C–E shows the RMSD of S1 domain of chains A, B and C at different temperatures. The Spike glycoprotein comprises of homotrimeric chain. Hence, to nullify the signal to noise ratio of individual chains, we also plotted average RMSD of the three chains at different temperatures (Figure 2F). The figure clearly shows that at 30 and 40°C, the RMSD is higher (∼0.5 nm) when compared to the average RMSD at 10, 20, and 50°C (∼0.42 nm). The above data indicates that the protein chains, especially the S1 domains are quite flexible around the temperatures of 30–40°C in comparison to low temperatures of 10°C or high 50°C of simulation temperature. Irrespective of the presence of the bilayer membrane, at different temperature conditions, the stalk of the Spike protein remains stable.

### Domain Flexibility of S1 Is More Pronounced

**FIGURE 3:**
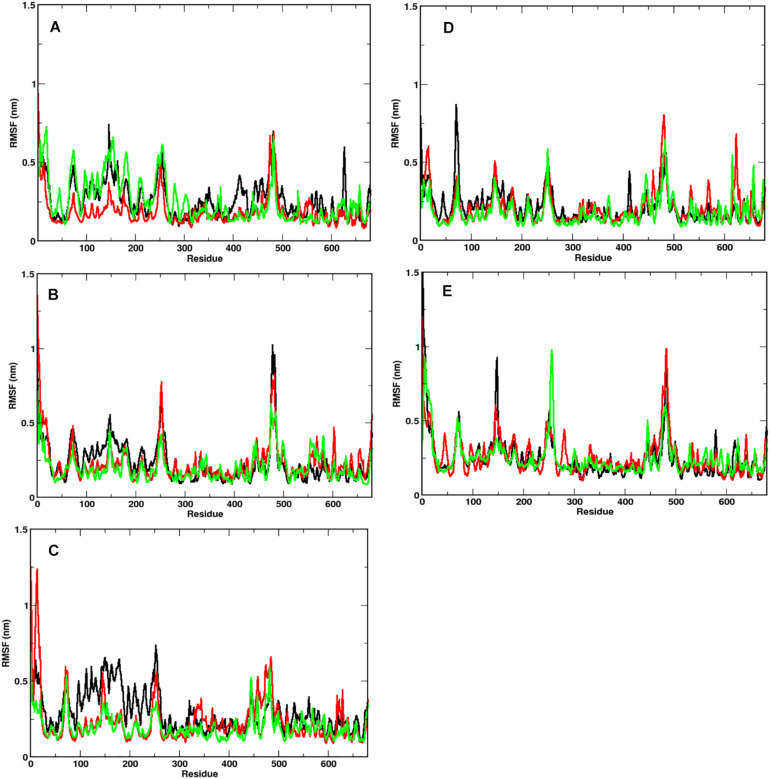
Fluctuation of CA of individual chains at different temperatures. RMSF of the CA atoms of the S1 domain for chains A (in black), B (in red), and C (in green) is shown. At temperatures **(A)** 10°C, **(B)** 20°C, **(C)** 30°C, **(D)** 40°C, and **(E)** 50°C, N-terminal domain (residues 1–333) have higher mobility than the receptor binding domain (residues 334–680).

In order to identify the region on the Spike protein that causes the deviations in RMSDs, we plotted the root mean square fluctuation (RMSF) of CA atoms of both S1 and S2 domains separately (Figure 3) at different temperatures. Each plot shows the RMSF of each individual chain at different temperatures. The RMSF of individual chains of S1 domain at different temperatures show that the residues ranging from 1 to 333 which constitute the N-Terminal Domain (NTD) of S1, show greater fluctuations compared to the Receptor binding Domain (RBD) ranging from residues 334 to 680. In the NTD, three distinct peaks could be seen, viz:- residues 85–90, 100–200, and 240–260. The first peak in the NTD was observed around residues 85–90 (β4–β5), which is a loop directed inwards to the S2 domain (Supplementary Figure S2). The peak was found to be highest in chain A at 40°C (∼0.8 nm), however, at other temperatures all the chains have approximately 0.5 nm RMS fluctuation of its CA atoms. The residues 100–200 constitute the solvent exposed β sheet (β6–β12) of the NTD of S1 domain (Supplementary Figure S2). The crystal structure (PDB: 6VXX) had shown as many as three glycosylated groups adjacent to this region of the protein (Supplementary Figure S3 and Berman et al., 2000). The residues 240–260 are solvent exposed loop around β14–β15. No glycan binding sites were observed in the crystal structure. The RBD domain consists of a receptor binding motif (RBM) ranging from residues 458 to 506 that show flexibility in all the systems. The lowest flexibility was observed at 10°C. At 30°C, the peaks were found for a wider range of residues. This indicates differential flexibility of the RBM at different temperatures. Since, the RBM is involved directly in binding to the ACE2 human receptor; its altered behavior at different temperatures would affect the protein-protein interaction. However, the average of the root mean square fluctuation of the three chains at different temperatures exhibit similar level of fluctuations (Supplementary Figure S2B). This indicates that although, movements of individual chains vary, the overall protein structure does not undergo major changes at different temperatures.

The RMSF of S2 domain on the other hand shows marked stability compared to domain S1 (Supplementary Figure S4). This is in good agreement to our earlier observations of the RMSD of the S2 domain. Since it is a triple helical coil, the coiled-coil motif of the S2 domain which is further supported by three shorter helices supports domain stability (Walls et al., 2016). However, the C-terminal residues 1,125–1,273 show greater flexibility compared to the rest of the domain. It should be noted that the C-terminal region of the Spike glycoprotein is exposed toward the inner side of the envelope bilayer and does not participate in the interchain interactions. It also has a more relaxed packing compared to the rest of the S2 (Berman et al., 2000; Guillén et al., 2005).

### NTD of the Spike Protein Could Act as a Receptor Binding Site

The NTD is relatively more exposed to solvents and more susceptible to external environmental conditions. However, unlike RBD, the NTD doesn’t have a defined open or closed conformation. The coronavirus NTD is composed of three layered beta-sheet sandwich with 7, 3 and 6 antiparallel β strands in each layer making it a total of 16 beta stranded sheet with 5 prominent β hairpin loops (Supplementary Figure S5). The crystal structures of Mouse Hepatitis Coronavirus (MHC) Spike protein and its receptor shows that the β1 and β6 of the NTD are the binding motif for CECAM1a protein (Shang et al., 2020). However, unlike the MHC NTD, the arrangement of strands in SARS-CoV-2 is in opposite direction. The upper layer of the beta sandwich is composed of beta strands β4, β6, β7, β8, β9, β10, β14 (Supplementary Figure S4). The three prominent regions which are exposed to the solvent and capable of interacting with potential receptors are regions N-terminal β strand, β8–β9, β9–β10, and β14–β15 loop.

**FIGURE 4:**
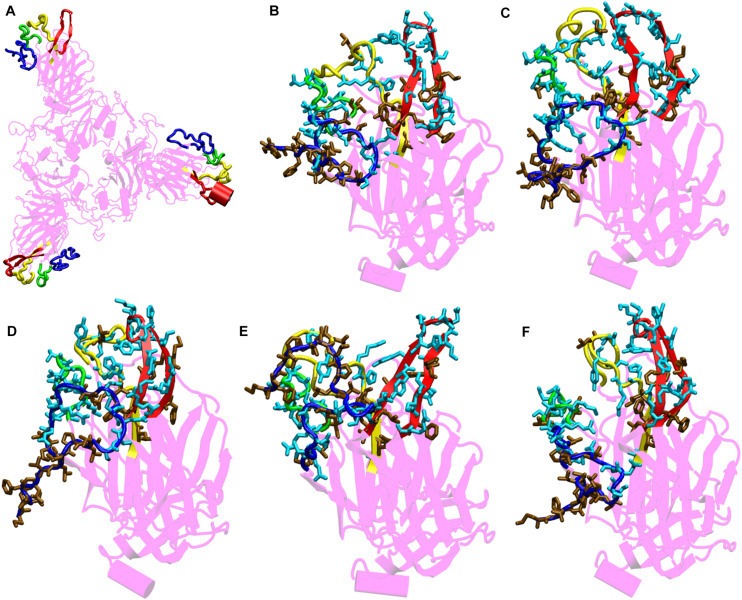
Structures of the N-terminal domain of Spike protein after 200 ns of simulation showing the relative orientation of solvent exposed loops. **(A)** The solvent exposed loops of NTD; the N-terminal β strand, β8–β9, β9–β10, and β14–β15 are shown in red, blue, green and yellow colors, respectively. Time-averaged conformation of N- terminal domain of SARS-CoV-2 Spike protein at, **(B)** 10°C, **(C)** 20°C, **(D)** 30°C, **(E)** 40°C, and **(F)** 50°C showing the relative orientation of the polar and hydrophobic residues. The residues are shown in licorice. Polar resides are colored in light blue and hydrophobic in brown colors, respectively.

Comparison of the NTD at different temperatures (Figure 4) show differential arrangement of the solvent exposed loops. The loops are formed by residues from N-terminal β strand, β8–β9, β9–β10, and β14–β15. The time averaged conformation of the loops after 200 ns of simulation show that the loops are oriented close to each other at temperatures 10–30°C, however at 40 and 50°C, they move farther away from each other. Moreover, comparison between Bovine coronavirus and Bovine hemagglutinin-esterase enzyme indicated close evolutionary link between the virus and the host proteins, which could facilitate attachment in the host cells (Li et al., 2013). Since, there was a similarity of NTD with the Ehprin A proteins (that binds to the Ephrin A receptors) we compared the residues involved in protein-protein interaction in the crystal structure of the human EphA4 ectodomain in complex with human Ephrin A5 for comparison (PDB ID: 4BKA) (Supplementary Figure S6). There are three salt bridges and seven hydrogen bonds between the Ephrin protein and its receptor. Moreover, it can be clearly seen that the NTD loops host a large number of polar residues (Figure 4). These residues form a stable motif at temperatures 10–30°C, primarily due to the stability between the loops. At 40 and 50°C, hydrophobic patch from N-terminal β strand is exposed toward the solvent. The polar residues from β9–β10 to β14–β15 move away from the N-terminal β strand and the β8–β9 loop, reducing the possibility of protein-protein interaction. Hence, a strong possibility exists for the NTD to act as a protein binding site at lower temperature ranges. There is also an uncanny correlation between the prevailing literature where scientists claim the virus affecting different parts of the human body and the similarity of NTD with human proteins (Gordon et al., 2020; Zaim et al., 2020).

From the bioinformatics and structural analyses (Figure 4 and Supplementary Figure S6), we observed that the NTD not only acts as a glycan binding site but can also as a site for binding of several human proteins. The motif formed out of several polar residues on the solvent exposed loops at 10–30°C could form salt-bridges and hydrogen bonds with partner proteins. At higher temperatures, the propensity of forming such interactions would be lost owing to the differential orientation of the loops. Nonetheless, the NTD could act as a possible target for development of vaccines and inhibitors. Similar vaccines developed against the NTD of Spike protein in mice, had earlier shown that NTD could act as a potential therapeutic target (Coleman et al., 2014; Jiaming et al., 2017).

### The RBD Behaves Differently at Higher Temperatures

The receptor binding domain (RBD) of the Spike glycoprotein is a potential target for vaccine and drug development (He et al., 2004; Tai et al., 2020). It is highly conserved among the human coronaviruses and binds to ACE2 receptor present on the lung tissues (Li, 2016). Residues 458–506 of the RBD domain comprises of the receptor binding motif (RBM). The RBM has 8 residues which are identical and 5 residues with similar biochemical properties between SARS, MERS and SARS-COV-2. This conserved region primarily interacts with the ACE2 receptor and hence, often scientists target the RBD domain of for developing therapeutic agents (He et al., 2004; Li, 2015; Tai et al., 2020). Earlier in Figure 2, we saw that the RBD domain spanning from residues 333–680 shows higher stability when compared to the NTD of the S1 domain at different range of temperatures.

**FIGURE 5:**
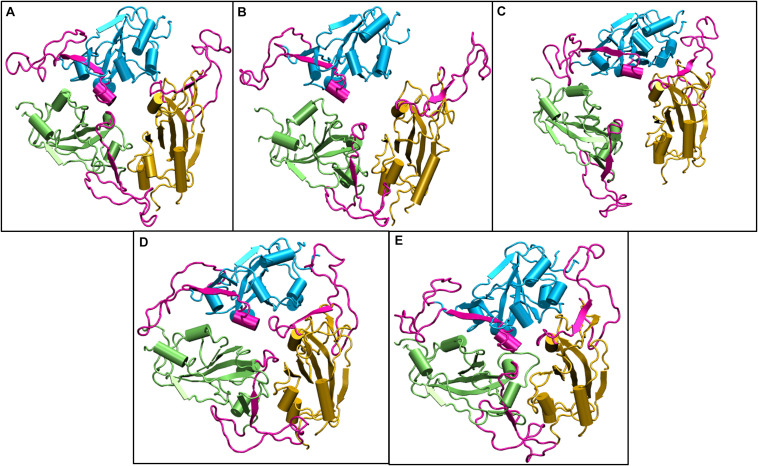
Structures of the receptor binding domain of Spike protein after 200 ns of simulation at different temperatures exhibit diverse structural dynamics. Time-averaged conformations of RBD of SARS-CoV-2 Spike protein at, **(A)** 10°C, **(B)** 20°C, **(C)** 30°C, **(D)** 40°C, and **(E)** 50°C. The three chains are colored in lime, cyan, and orange. The receptor binding motif (shown in magenta) is oriented in a confined conformation at higher temperatures.

We compared the time averaged conformation of the RBD generated from the last 10ns of the simulation time at different temperatures (Figure 5). The core β pleated sheet was very stable demonstrating no lack of secondary structures at higher temperatures. However, the RBM motif (highlighted in magenta in Figure 5) shows a very dynamic conformation across different temperature ranges. The dynamics was more pronounced at 10, 20, and 30°C whereas at 40 and 50°C of temperature, the RBM had a more confined conformation. The RBD flexibility was more apparent at 20 and 30°C where the three chains moved further away from each other. However, a tighter and well packed structure was found for the protein at 50°C. The figures suggest that although residue wise movements in RBD were not visible in RMSF (Figure 2), the RBD domains and motifs show intrinsic flexibility along particular temperature ranges. Previous studies have indicated that the RBD domain can adopt either an open or a closed conformation in the virus (Walls et al., 2020). We compared the conformation of the Spikeprotein-ACE2 crystal structure and found that in the open conformation, the RBD exposes its RBM residues Phe456, Ala475, Phe486, Asn487, Tyr489, Gln493, Gly496, Gln498, Thr500, Asn501, Gly502, and Tyr505 to facilitate the binding of the ACE2 receptors. It is fascinating to see that at 40°C and more interestingly at 50°C, the RBM motif is in a closed loop conformation and very compact which hinders its association with the partner proteins.

### Spike Protein Adopts a Closed Conformation at Higher Temperature

Principal Component Analysis (PCA) was carried out to study the different conformations generated during the simulations. Principal components help us in identifying the most essential motions in complex systems (Berendsena and Haywardb, 2000; Meyer et al., 2006). To get a more pronounced picture, we studied the principal components of only the S1 domain. As seen in Supplementary Figure S6, the first 5 eigenvectors capture nearly 50% of the dynamics of the protein. We then went on to project the principal components along the first and second eigenvectors (Supplementary Figure S6B). The X axis represents the PC1 where maximum fluctuations could be seen. It can be seen that the data fluctuates between -18 to 15 in X axis and -7 and 12 in Y axis. At lower temperatures (10 and 20°C), the fluctuations varied between -8 and 12 at X axis, at 30°C the fluctuations were relatively higher varying from -10 to 15. However, at increased temperature of 40°C the data varied between -12 and 8 drifting more toward the left. At 50°C it further shifted toward the left varying from -18 to 4. In the Y axis at temperatures 10, 20, and 30°C, the data varied between -9 and 10. At 40°C it varied from -12 to 9. Surprisingly at 50°C, it only fluctuated between -10 and 5, indicating marked restrictions in movements. This clearly indicates larges conformational changes in the S1 domain at higher temperatures. The flexibility of the protein was found to have reduced with the increase in temperature.

**FIGURE 6:**
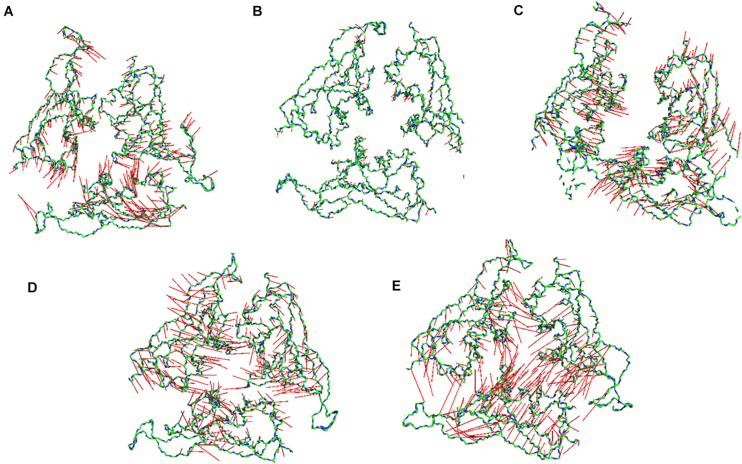
Porcupine plots of the RBD of SARS-CoV-2 Spike protein. Porcupine plots generated from the extreme conformations of the RBD of the Spike protein at **(A)** 10°C, **(B)** 20°C, **(C)** 30°C, **(D)** 40°C, and **(E)** 50°C showing difference in dynamics of the protein at different temperature. The arrows are colored in red and the protein backbone is shown as sticks colored by CPK. More arrows are pointed toward the protein core at 40 and 50°C implying a closed conformation.

Subsequently, we went on to check the extreme movements of the CA atoms of the S1 domain along the first principal component (Figure 6). The figure clearly shows that the arrows at 10, 20, and 30°C the three chains do not point toward each other, although the dynamics was high at 10 and 30°C. This would facilitate a more open conformation. The degree of movement was found to be more at 30°C which corresponds well with Figure 5 as described above. Surprisingly, at 40 and 50°C, opposite domain movements were observed. The arrows point toward each other in these systems. Additionally, the three chains come close to each other and reduce the accessible area.

**FIGURE 7:**
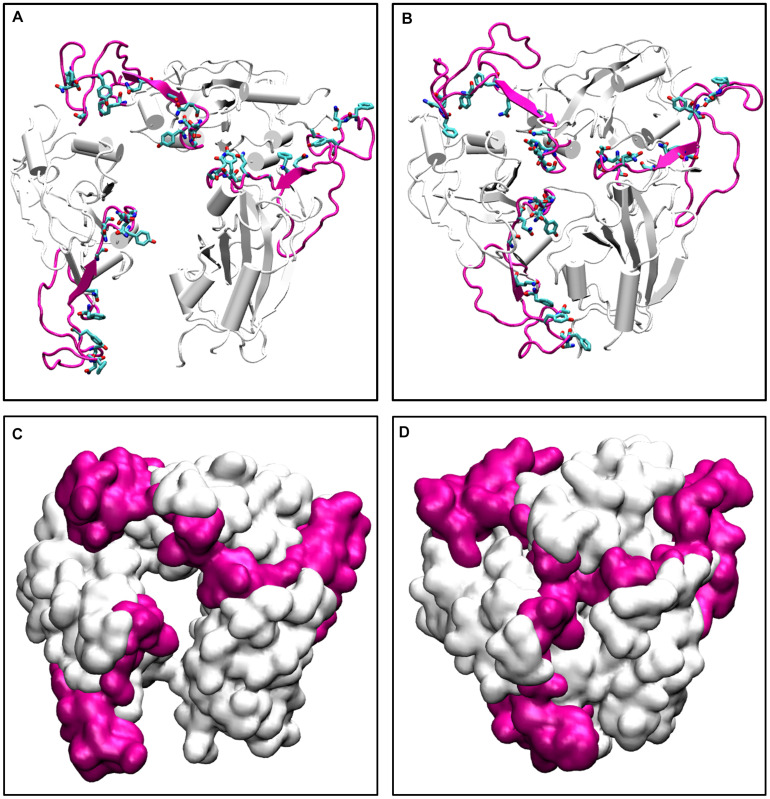
The conformations of receptor binding motif. The time averaged structures of the Spike protein showing the open and closed conformations of the receptor binding domain at **(A)** 30°C and **(B)** 70°C. The residues on the Receptor binding motif (shown as sticks and colored by CPK) are buried at the interchain interface at high temperatures but readily exposes its residues at 30°C. **(C,D)** show the open and closed conformations of the receptor binding domain at **(A)** 30°C and **(B)** 70°C in surf mode. The receptor binding motif is colored in magenta and the receptor binding domain is shown in white.

To further validate our findings, we ran another simulation of the Spike protein at a higher temperature of 70°C. After 100 ns of simulation, we found that significant similarity between the closed conformation observed at 50°C and the conformation at 70°C. The RBM residues, specifically Phe456, Ala475, Phe486, Asn487, Tyr489, Gln493, Gly496, Gln498, Thr500, Asn501, Gly502, and Tyr505 were found to be clearly buried between the interchain subunits at 70°C (Figure 7 and Supplementary Figure S7). However, when compared to the orientation at 30°C the residues are directed toward the solvent. Thus, the reason for very stable RMSD observed in Figure 1, is largely due to the confined architecture of the receptor binding domain at 50°C and higher temperatures. The unavailability of RBM residues to bind to ACE2 receptor would nonetheless destabilize virus-protein interactions at higher temperatures.

**FIGURE 8:**
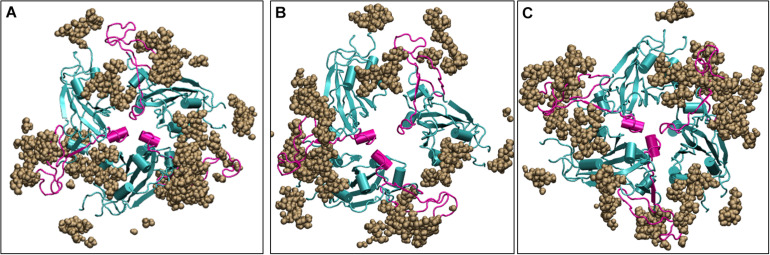
Structures of the receptor binding domain of glycan bound Spike protein after 200 ns of simulation displays similar behavior to that protein in the absence of glycans. Time-averaged conformations of RBD of SARS-CoV-2 Spike protein at, **(A)** 10°C, **(B)** 30°C, and **(C)** 50°C. The glycan moieties are colored in brown and shown in Vander Waal’s representation. Protein is shown in cyan color and the RBM motif in magenta. RBD, receptor binding domain; NTD, N –terminal domain; RBM, receptor binding motif; ACE2, angiotensin converting enzyme 2.

Since the Spike protein is surrounded by glycans, it is of utmost importance to know if the carbohydrates cause change in the dynamics of the Spike protein. We ran three additional simulations with the glycans one at 10°C, one at 30°C and one at high temperature of 50°C for 200 ns. We found that irrespective of the presence of carbohydrates, the dynamics of the protein remains similar although the magnitude is diminished due to the large number of glycan chains. Figure 8 shows the average conformation of the Spike protein RBD domain in the presence of carbohydrate molecules. At lower temperature of 10 and 30°C, the carbohydrate moieties are interspersed between the protein chains and the complex is open, similar to the structure without carbohydrates (Figure 5). The closed conformation at 50°C can be clearly observed in Figure 8, where now the carbohydrates appear to be surrounding the protein while the protein closes its RBM. Thus, although carbohydrates are very important for the immunogenicity of the protein, the temperature dependent conformation of the spike glycoprotein is largely due to the protein dynamics.

Thus, although the RBM stays largely in open conformation state, surprisingly, from around the temperature of 40°C, a closed conformation of the motif was observed. In this conformation, the RBM from the three chains come very close to each other sealing the visibility of the trimeric pore. At temperatures >50°C, the Spike RBM is completely closed (Figure 7). The closing of the RBM buries the receptor binding residues inside trimer abolishing the possibility of contacts with the ACE2 receptor and making the Spike protein inactive. Our results clearly show that the activity of the Spike protein is dependent on the external temperatures where a higher temperature renders it completely inactive.

## Conclusion

The SARS-CoV-2 has severely affected the human population with large number of infected individuals around world. The propensity of virus to survive in cold and dry climatic conditions have been speculated by researchers and supported by the statistical evidence from earlier SARS epidemic of 2002. However, it is still unclear how the virus undergoes changes at the structural level in different environments. The Spike protein of the virus helps in the attachment and entry of the coronaviruses inside the host cells. It exists as a homotrimer and is partly exposed to the outer environment and partly immersed inside the lipid bilayer of the viral envelope. Here, we studied the differential response of the Spike protein at different temperature conditions.

Our results show that the S2 transmembrane domain remains stable even without the bilayer membrane, whereas the solvent exposed S1 domain is quite flexible. Moreover, the S1 comprises of two subdomains, namely N-terminal domain (NTD) and the receptor binding domain (RBD). The simulations results show that the RBD is relatively less mobile. Its flexibility is limited only to the receptor binding motif or RBM which interacts with the Angiotensin Converting enzyme 2 (ACE2), its human receptor. However, the NTD was found to be quite mobile.

Although, the NTD doesn’t directly interact with the ACE2 receptor in humans, it has been found to bind to receptors in other mammals (Humphrey et al., 1996). The flexible NTD hosts a large number of charged residues on the top layer of its tri-layered beta sandwich architecture. However, at 40–50°C of temperature, the polar residues were found to be less solvent exposed. The similarity of the NTD sequence with the several human receptors such as Ephrins, Briakunumab, anti-TSLP, etc. indicated a possibility of the subdomain to be involved in binding to alternate human proteins.

The RBM present on the RBD is very crucial in initial protein-protein interaction between the host and virus. We found that this domain is largely in an open conformation which enables receptor binding at lower temperatures. Surprisingly, from ∼40°C, a closed conformation of the motif was observed. In this conformation, the RBM from the three chains come very close to each other sealing the visibility of the trimeric pore. At temperatures >50°C, the Spike RBM is completely closed. This was also evident from the dynamics obtained from principle component analysis. The closing of the RBM buries the receptor binding residues inside the trimer abolishing the possibility of contacts with the ACE2 receptor and making the Spike protein inactive. The same phenomena was observed in the presence of glycan moieties.

Our results have shown for the first time that the Spike protein has the possibility to stay in an active and inactive state based on the external temperature. They corroborate very well with the experimental observations and observations from simulation studies which talks about thermal stability and cold-induced destabilization of the protein (Edwards et al., 2020; He et al., 2020; Ou et al., 2020; Xiong et al., 2020). Moreover, since no visible loss of secondary structure was observed at higher temperatures (Supplementary Figure S8), it would be interesting to know if the conformational change is reversible in nature. Nevertheless, this work would prove very beneficial in the development of vaccines as well as development of therapeutic strategies that target not only the receptor binding domain but also the N-terminal domain of the Spike protein.

## Data Availability Statement

The raw data supporting the conclusions of this article will be made available by the authors, without undue reservation, to any qualified researcher.

## Author Contributions

SR and KK conceived and designed the experiments. SR performed the experiments, analyzed the data, contributed reagents, materials, analysis tools, and wrote the manuscript. All authors contributed to the article and approved the submitted version.

## Conflict of Interest

The authors declare that the research was conducted in the absence of any commercial or financial relationships that could be construed as a potential conflict of interest.
