# Supplementary material for: Investigation of the Effect of Temperature on the Structure of SARS-CoV-2 Spike Protein by Molecular Dynamics Simulations
Source: Front Mol Biosci. 2020 Oct 16;7:583523. doi: 10.3389/fmolb.2020.583523 (PMC7596554; doi:10.3389/fmolb.2020.583523)
Supplement: Supplementary file 1 [file Data_Sheet_1.pdf]

Investigation of the effect of temperature on the structure of SARS-Cov-2 Spike Protein by Molecular Dynamics Simulations

Soumya Lipsa Rath[1*] and Kishant Kumar[2]
[1]Department of Biotechnology, National Institute of Technology Warangal (NITW), Telangana, India, 506004
[2] Department of Chemical Engineering, National Institute of Technology Warangal (NITW), Telangana, India, 506004

*** Correspondence:**
Corresponding Author
slrath@nitw.ac.in

Table S1. List of systems studied

Serial No.	Temperature (in ℃)	Simulation Time (ns)
1	10	200
2	20	200
3	30	200
4	40	200
5	50	200
6	70	100
7	10 (protein + glycan)	200
8	30 (protein + glycan)	200
9	50 (protein + glycan)	200

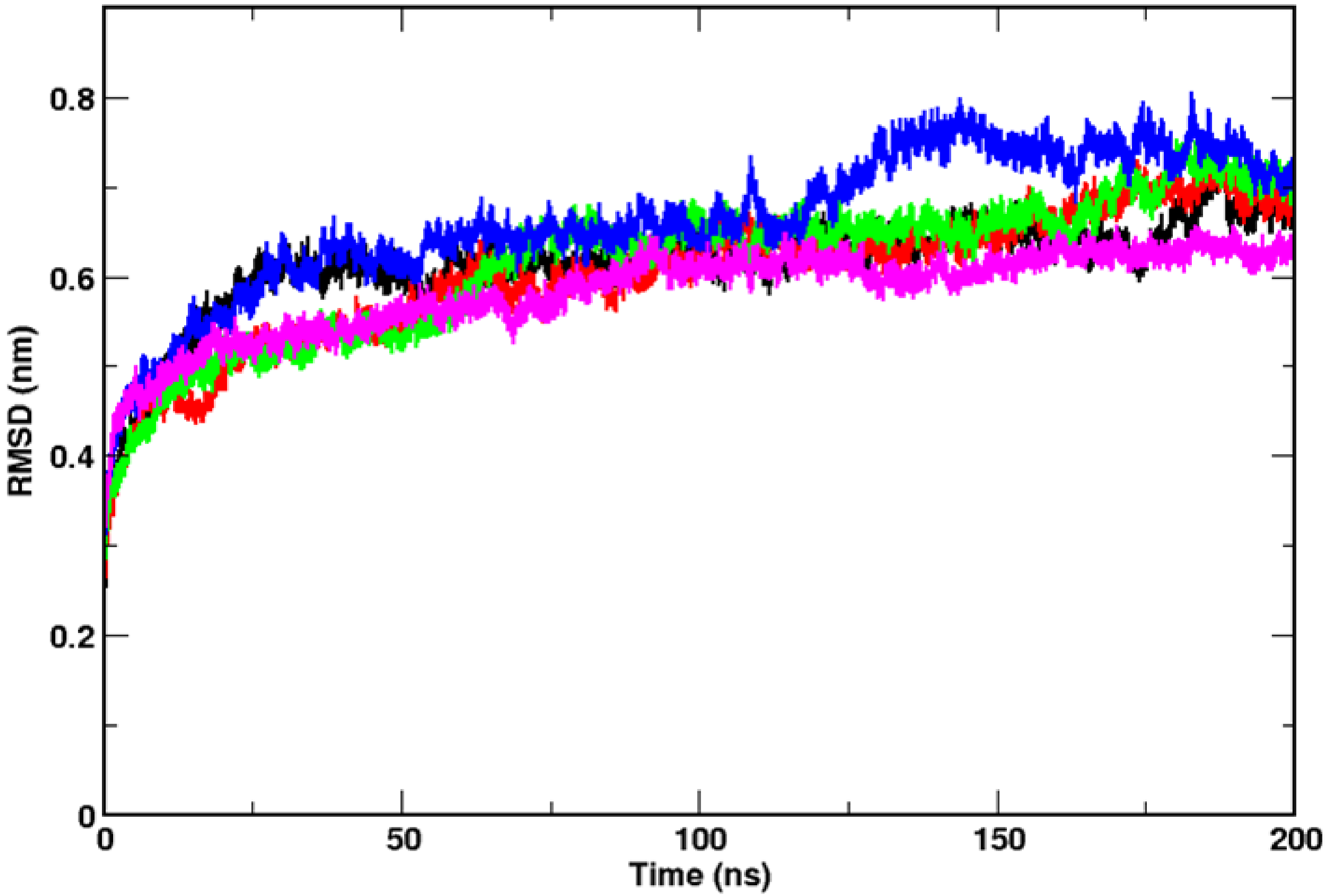

Figure S1. Stability of the Spike protein across all temperatures. RMSDs of Spike glycoprotein at 10 ℃(black), 20 ℃ (red), 30 ℃ (green), 40 ℃ (blue) and 50 ℃ (magenta) during 200ns of classical MD simulations showing stability of the simulations

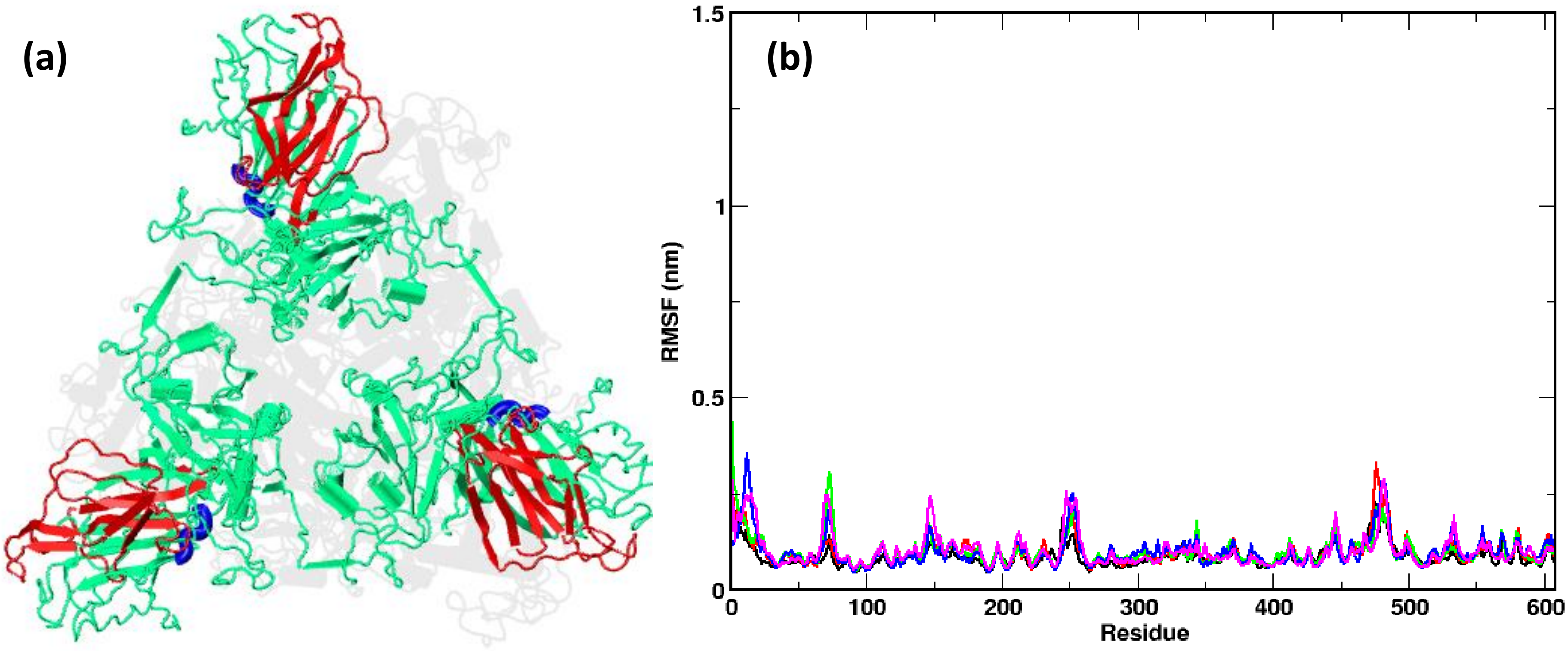

Figure S2. Highly fluctuating regions in the N-Terminal Domain (a) Structure showing the S1 domain from top, highlighting the regions where peaks were observed in RMSF. The β4-β5 loop is shown in blue and the solvent exposed β6-β12 loop is shown in red. (b) The average RMSF of the three chains of S1 domain however show very less structural change.

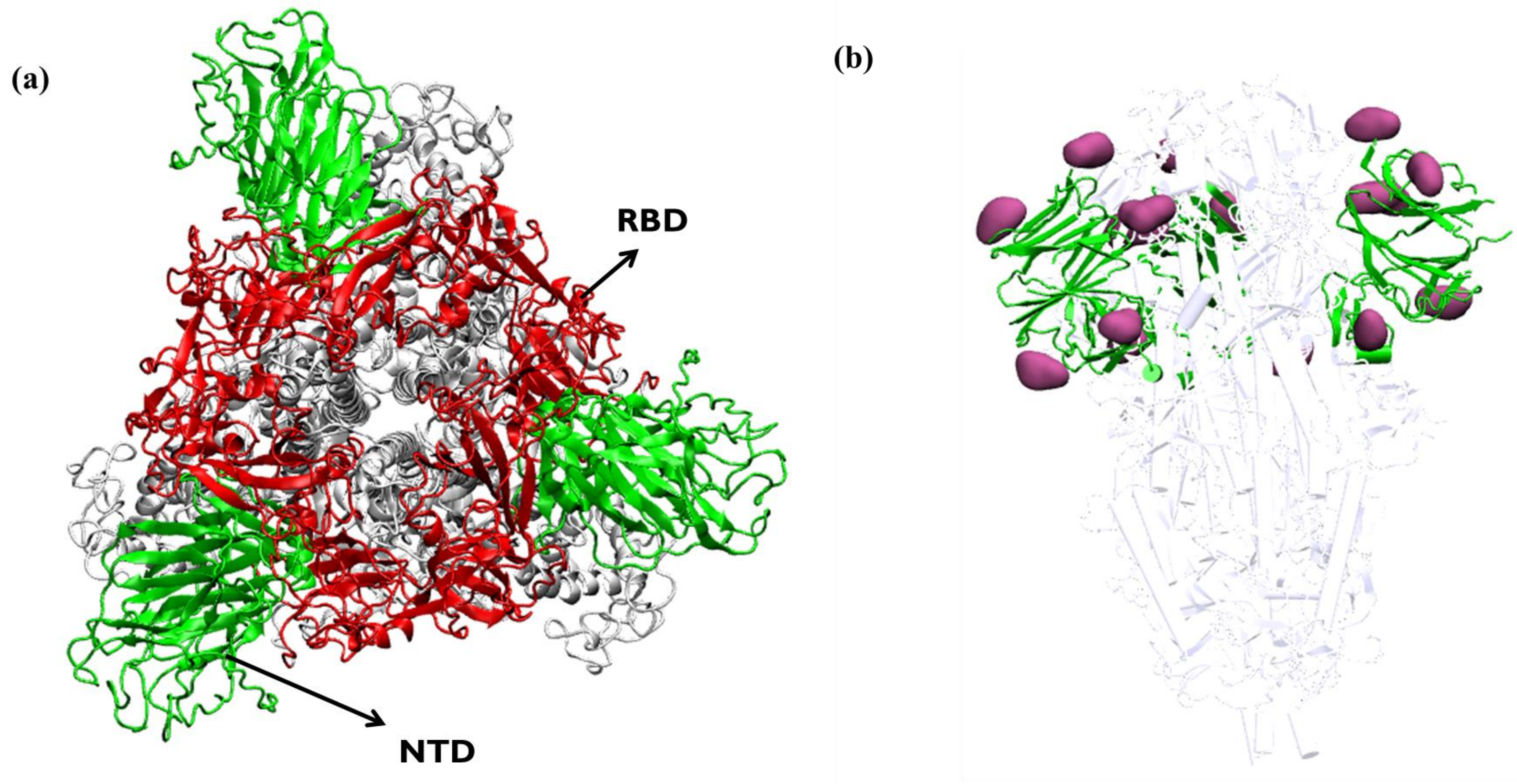

Figure S3. The structure of S1 domain. (a) Structure of S1 domain highlighting the N-Terminal Domain (in green) and Receptor Binding Domain (in red). (b) The orientation of N-acetyl glucosamine (NAG) residues around N-Terminal Domain in the crystal structure of Spike protein (PDB: 6VXX). The NAG residues are shown in surf mode and colored in pink, the N-Terminal Domain is shown in green

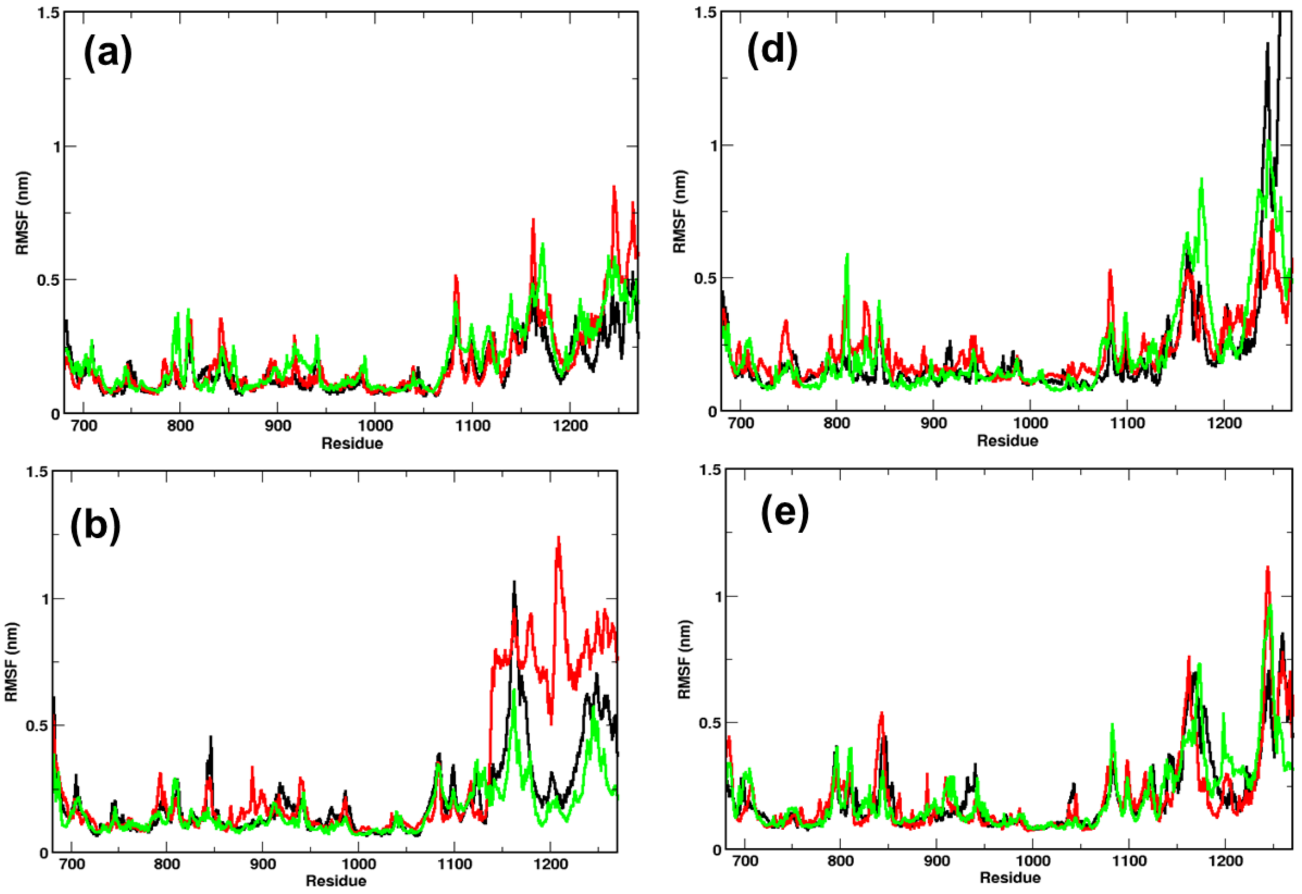

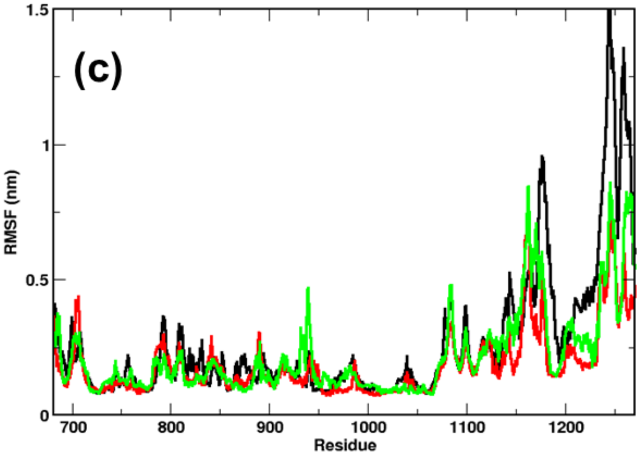

Figure S4. **Fluctuation of CA of individual chains at different temperatures**. RMSFs of the CA residues of S2 domain showing stability in residues spanning the transmembrane region. Fluctuations of chain A (in black), B (in red) and C (in green) are shown for temperatures (a) 10 ℃, (b) 20 ℃ , (c) 30 ℃ , (d) 40 ℃ and (e) 50 ℃

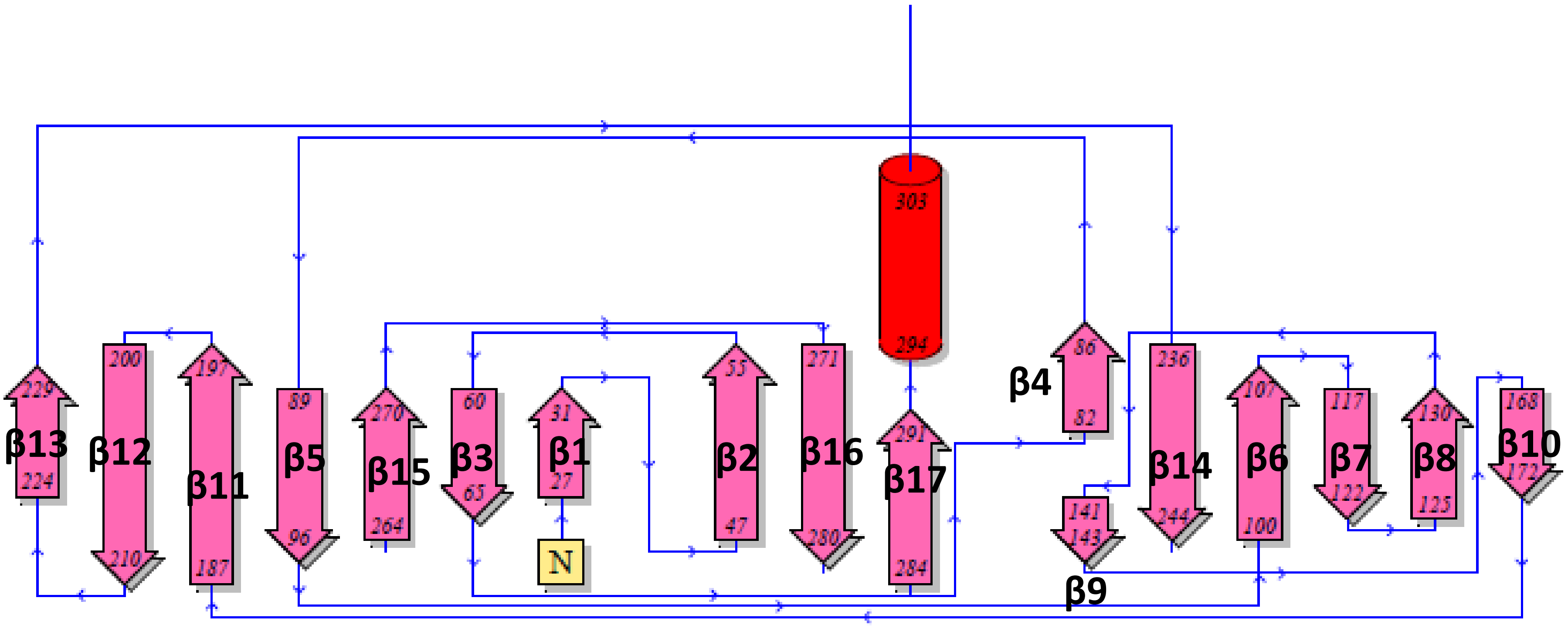

Figure S5. Secondary structure of the N-Terminal Domain. Topology of the N-Terminal Domain showing the three distinct layers of β sheet. β strands are colored in pink and α helix in red. The beginning and end residues are shown. The structure is generated from the pdbsum online server using the crystal structure of Spike protein (PDB ID: 6VXX)

(a) MGILPSPGMPALLSLVSLLSVLLMGCVAETGTQCVNLTTRTQLPPAYTNSFTRGVYYPDKVFRSSVLHSTQDLF
LPFFSNVTWFHAIHVSGTNGTKRFDNPVLPFNDGVYFASTEKSNIIRGWIFGTTLDSKTQSLLIVNNATNVVIKVC
EFQFCNDPFLGVYYHKNNKSWMESEFRVYSSANNCTFEYVSQPFLMDLEGKQGNFKNLREFVFKNIDGYFKIY
SKHTPINLVRDLPQGFSALEPLVDLPIGINITRFQTLLALHRSYLTPGDSSSGWTAGAAAYYVGYLQPRTFLLKY
NENGTITDAVDCALDPLSETKCTLKSFTVEKGIYQ

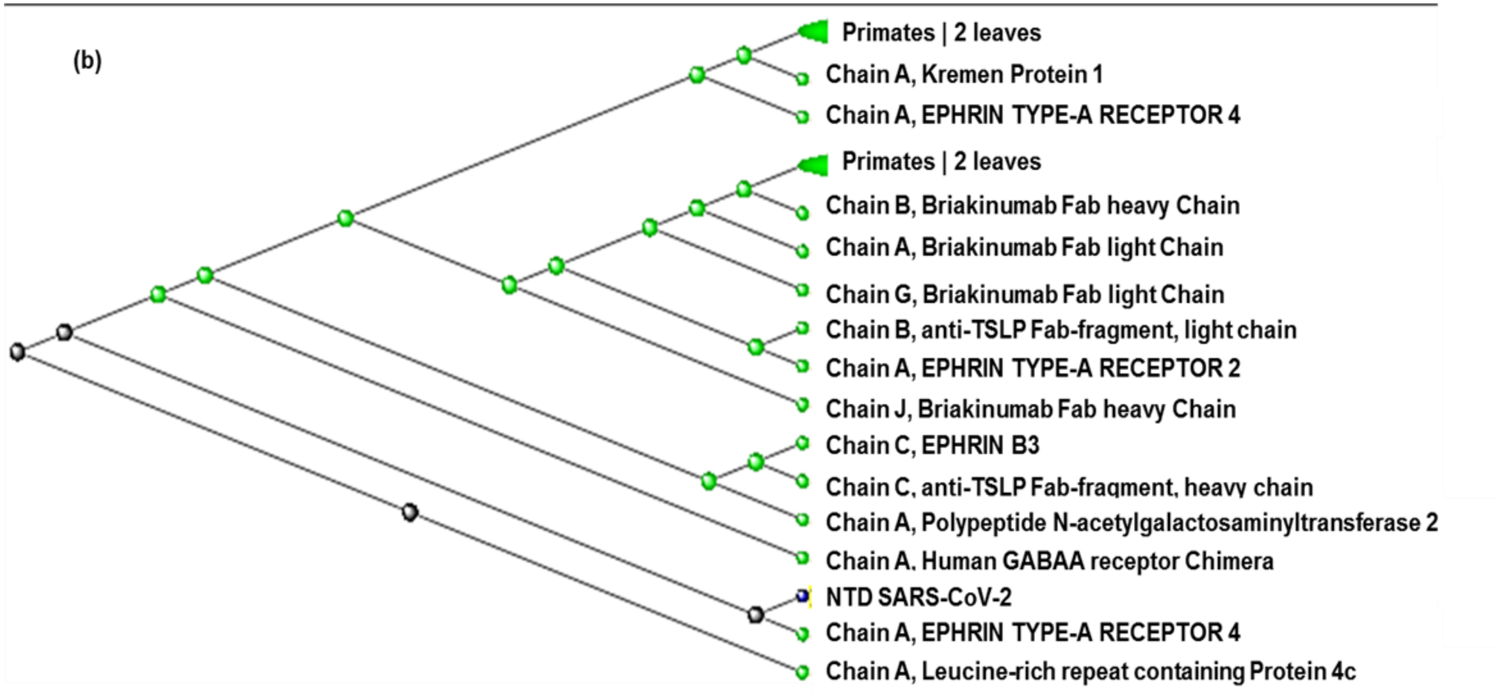

Figure S6: Similarity of NTD of SARS-CoV-2 with human proteins (a) The sequence of the N-Terminal Domain of SARS-CoV-2 obtained from crystal structure which was used for Multiple sequence alignment and constructing phylogenetic tree (b) The Phylogenetic tree constructed from the results of multiple sequence alignments of N-Terminal domain of S1 and sequences of human proteome.

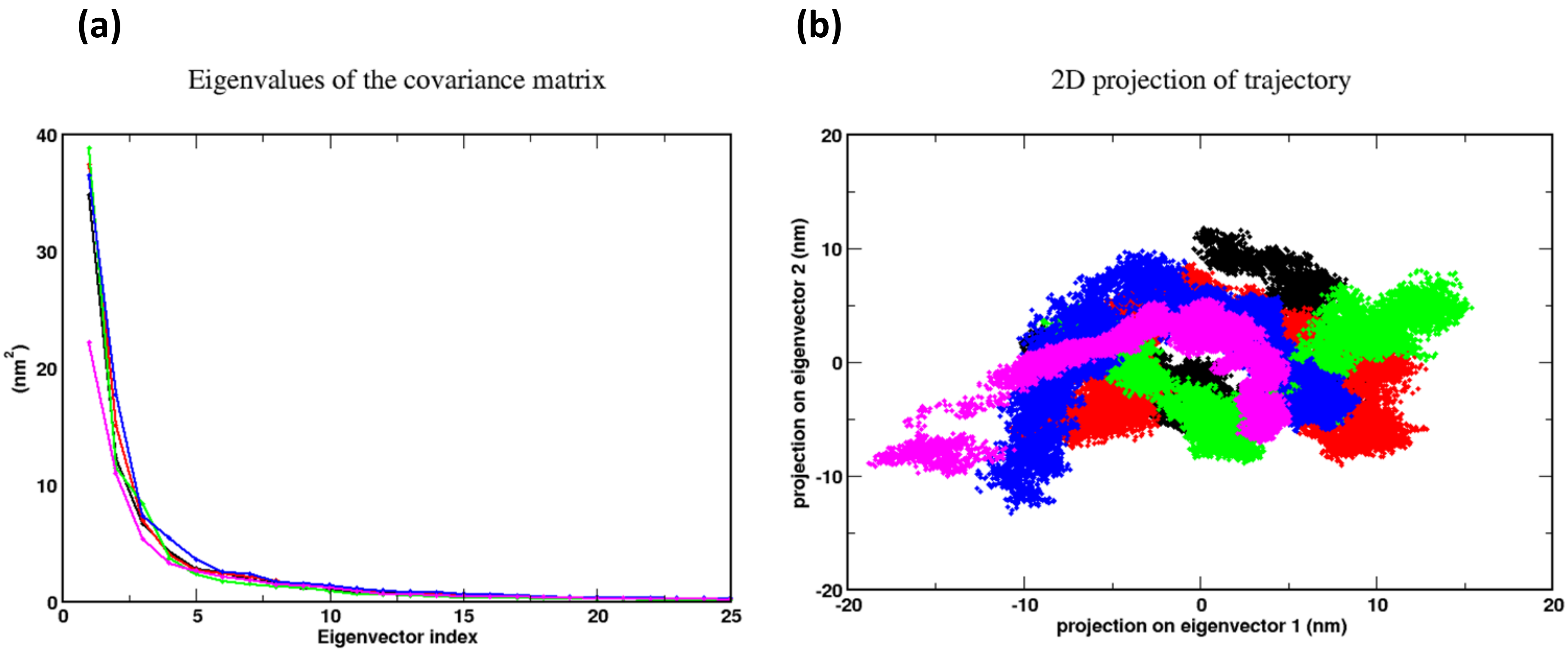

Figure S6. Eigenvalue profile and comparative sampling of essential motions in protein at different temperature. (A) Eigenvalues of the first 25 eigenvectors, derived from PCA analyses of the simulation trajectories are shown. (B) Two dimensional projection of the simulated structures on the plane constituted by the first two principal components, in all the complexes. Color code :10 ℃(black), 20 ℃ (red), 30 ℃ (green), 40 ℃ (blue) and 50 ℃ (magenta)

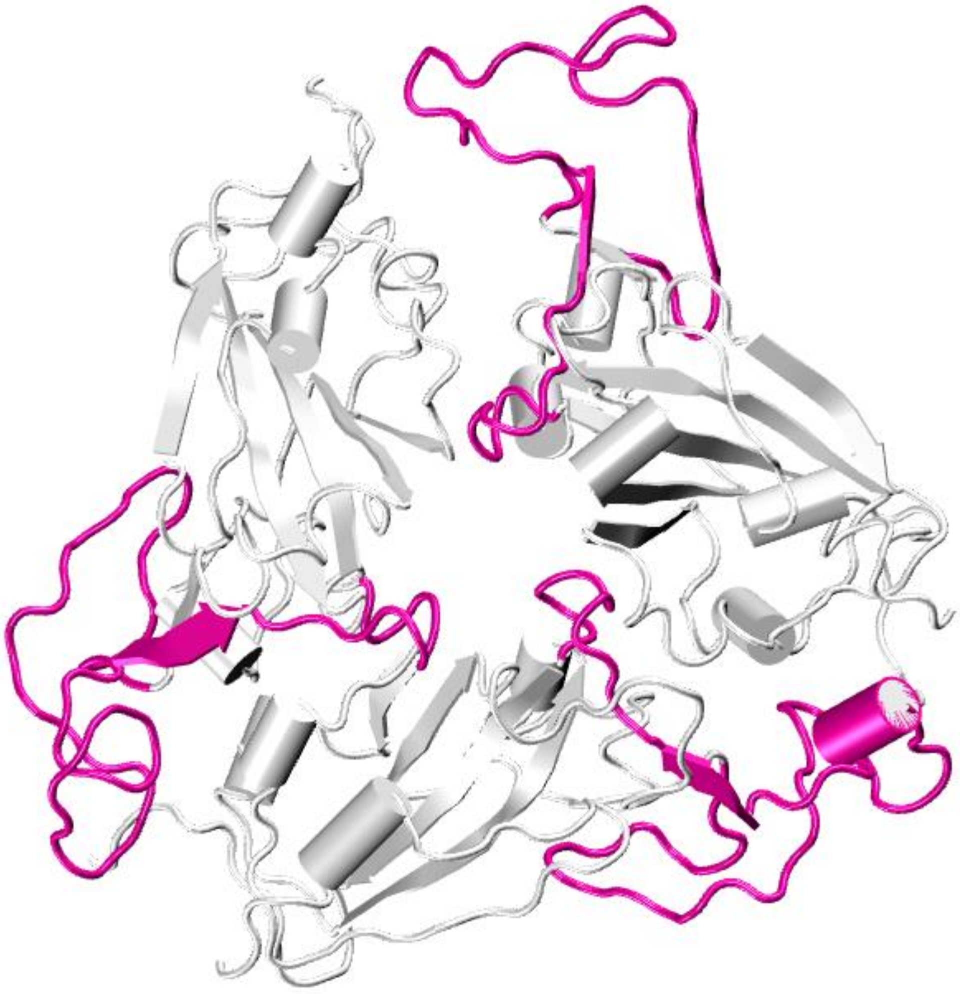

Figure S7. Spike protein receptor binding motif at high temperature. The time average conformation of the receptor binding motif highlighted in magenta at 70 ℃ after 100ns of simulation, showing the confined arrangement of loops

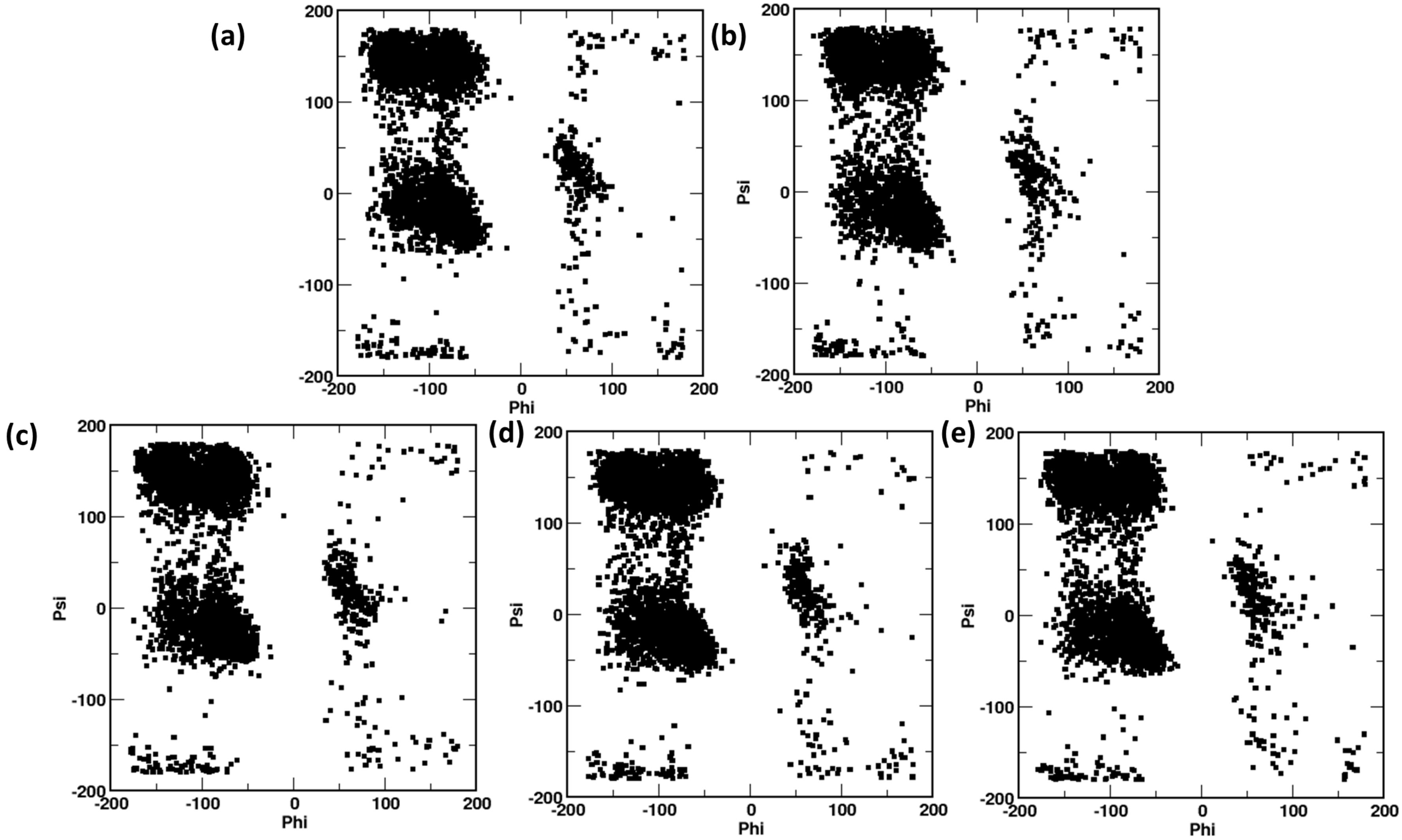

Figure S8. **Ramachandran Plot of the Spike glycoprotein at different temperatures.** Ramachandran plot of final conformation of the Spike glycoprotein at (a) 10 ℃, (b) 20 ℃, (c) 30 ℃, (d) 40 ℃ and (e) 50 ℃ showing no loss of the secondary structures at higher temperatures.
